# Artificial intelligence centric scientific research on COVID-19: an analysis based on scientometrics data

**DOI:** 10.1007/s11042-023-14642-4

**Published:** 2023-03-02

**Authors:** Amit K. Shukla, Taniya Seth, Pranab K. Muhuri

**Affiliations:** 1 Faculty of Information Technology, University of Jyväskylä, Box 35 (Agora), Jyväskylä, 40014 Finland; 2 Department of Computer Science, South Asian University, Akbar Bhawan, Chanakyapuri, New Delhi 110021 India

**Keywords:** Artificial intelligence, Bibliometric analysis, Computer science research, Coronavirus research, COVID-19, Publications, Research analysis

## Abstract

With the spread of the deadly coronavirus disease throughout the geographies of the globe, expertise from every field has been sought to fight the impact of the virus. The use of Artificial Intelligence (AI), especially, has been the center of attention due to its capability to produce trustworthy results in a reasonable time. As a result, AI centric based research on coronavirus (or COVID-19) has been receiving growing attention from different domains ranging from medicine, virology, and psychiatry etc. We present this comprehensive study that closely monitors the impact of the pandemic on global research activities related exclusively to AI. In this article, we produce highly informative insights pertaining to publications, such as the best articles, research areas, most productive and influential journals, authors, and institutions. Studies are made on top 50 most cited articles to identify the most influential AI subcategories. We also study the outcome of research from different geographic areas while identifying the research collaborations that have had an impact. This study also compares the outcome of research from the different countries around the globe and produces insights on the same.

## Introduction

**Fig. 1:**
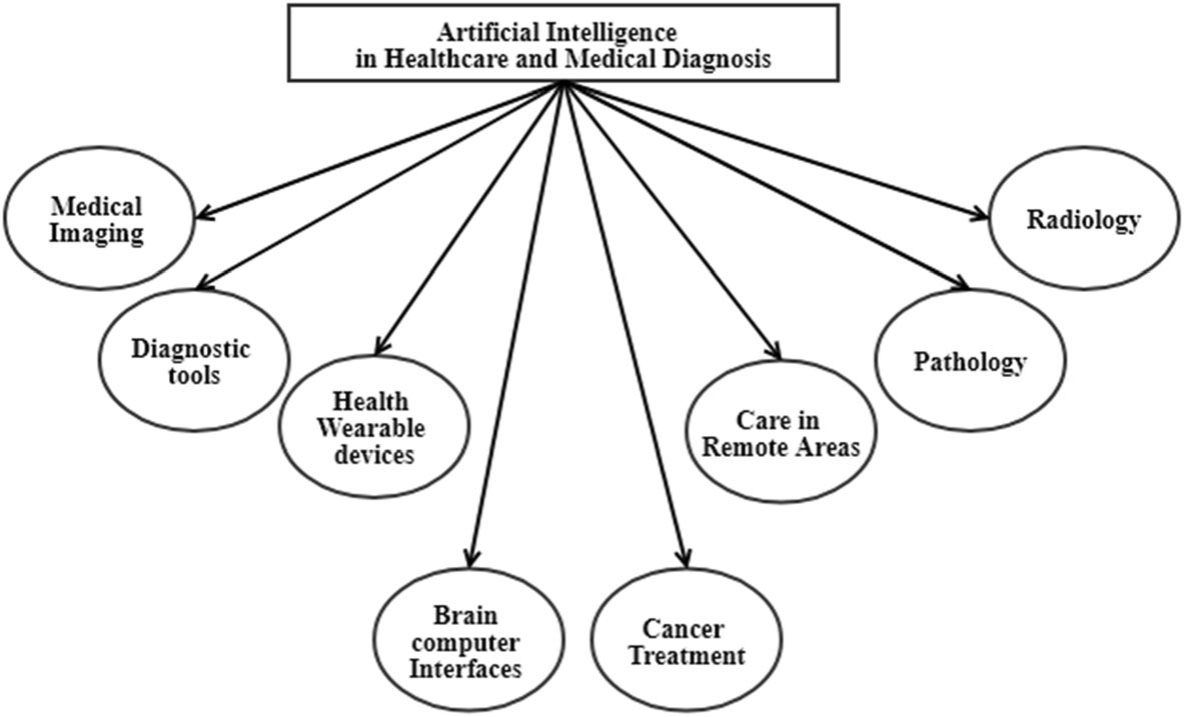
Applicative areas of AI in healthcare and medical diagnosis

The omnipresence of Artificial Intelligence (AI) within the last decade is magnificent considering its applicability in almost all the real-world contexts. AI is powered by data science and exploratory analysis (DS/DEA), machine learning (ML) and deep learning (DL) approaches, which have shown significant improvements in several domains such as cyber security [47], cancer treatment [10], clean energy [54], financial sector [29], global education [45], etc. Healthcare and medical diagnosis are the other crucial areas where AI has shown potential in analyzing big data sets. Whether it is analytics on patient information to provide accurate predictive analysis or discovering new drugs to cure novel diseases in a timely manner, AI has come a very long way. To visualize the overall picture, Fig. 1 sums up various sectors in the medical domain, where AI has brought revolution and has increased the efficiency of the outcomes. These classifications are just representative to provide the perspective of the effectiveness of AI in these diverse areas.

Such effectiveness of AI can be seen in assisting scientists in tackling the recent pandemic caused by the coronavirus also termed as COVID-19. As of August 15, 2021, a total of 201 million [86] cases of the novel coronavirus disease have been registered globally since the first case of the same was reported from China in December 2019 [82]. This figure of affected cases worldwide is a clear indication of how deadly the novel COVID-19 is. Due to the widespread impact of the virus, research activity related to COVID-19 has been of central importance to many researchers worldwide, in the hope of discovering a new drug to cure the disease, to obtain trends of active or affected cases, or to predict the occurrence of the disease in individuals. Since there was no immediate cure to this virus, the number of fatalities increased gradually worldwide. It was hence necessary to target this devastating pervasion with state-of-the-art AI tools that could help fight the battle with COVID-19. The computational and precise predictions from AI approaches are quite evident, as can be seen from the results in various real-world applications [32, 33, 62, 71, 74, 36].

Keeping an eye on this boost in AI centric based research on COVID-19, it is crucial to devise a systematic methodology to identify the sources of the most impactful research. With this paper, we aim to construct a bridge between the publications and interested researchers who would like to identify the most impactful research content as well as related information. Furthermore, a benchmark of current scientific publications would also be created through such a study, leading to more interest from researchers in various fields belonging to diverse institutions and geographies converging on this area.

Scientometric (also refereed as Bibliometric) analysis provides a systemic review format for studying research publications within a scientific domain. This includes analyses of the citation structure, best papers based on citation count, research areas, highly productive and influential journals, impactful authors, co-citation amongst authors, institutional participation, geographic contribution, bibliographic coupling amongst authors, countries, institutions, as well as that of journals etc. Such analyses have been performed on multiple domains till date, such as a journal specific study of publication and citations structure [57, 61, 95]. In addition to these, there are studies which provided the results of a scientometric analysis performed to gain insight into research areas such as the study of aggregation operators [11], industry 4.0 revolution [58], brain MRI research [18], multimedia big data [43], etc. Similarly in the medical domain, the scientific outputs were tracked within the topics of the Ebola outbreak [93], global malaria vaccine [35], and Dengue research [96]. A study akin to these was also performed in [3], pertaining to the Zika virus outbreak.

Some researchers in the past studied the research landscape related to COVID-19. In [67], Sahoo and Pandey evaluated the research performance of the overall pandemic due to COVID-19, based on scientometric indicators, where the literature data was obtained from Scopus. Colavizza et al. [25] also recently produced a scientometric overview of the CORD-19 dataset, which is regularly updated with medical related articles from Medline, PubMed, the WHO database, arXiv, bioRxiv and medRxiv. Authors hence presents a detailed literature study based on medical literature and the related dataset. Another such analysis was performed in [37] where in addition to a scientometric study, safety related research dimensions were also presented through a scoping review, which identified the different types of safety issues that attracted research. Similarly, scientometric analyses were also performed in [2, 52], focusing on research related to the coronaviruses from 1900 to 2020 and the overall statistics of the global research output respectively. Cunningham et al. [28] produced results from a scientometric analysis with the main aim of studying collaborations in the time of COVID-19. Apart from these, some researchers also considered the impact and hence-forth, the interest of COVID-19 research on specific areas such as endocrinology [7] and ophthalmology [44].

The literature also finds some traces of bibliometric/scientometric studies targeted on AI or related domains. Islam et al. [40] studies the initial bibliometric analysis of COVID-19 on AI. They included only 729 papers with only quantitative analysis of the data gathered from Web of Science (WoS). In a similar study, Wu et al. [89], studied about 1903 articles with the emphasis on visual knowledge map analysis. Abumalloh et al. [1] presented the systematic literature review of the computational approaches of medical image processing specific to the COVID-19. The study by Chicaiza et al. [20] covered the bibliometric analysis of DL related works only for the year 2020. Rodríguez-Rodríguez et al. [66] performed the scientometric analysis of the AI, ML, big data, and Internet of Things (IoT) approaches with applications on the pandemic. In this paper, we have obtained an in-depth knowledge about the current structure of publications related to AI centric research on COVID-19. A bibliometric analysis is presented of the scientific publication data available on the WoS database [24]. WoS is accredited with high-quality publications from high ranked journals and international conferences. The factors such as citation counts, number of publications, and their document types are considered. Furthermore, data related to the highly cited research on AI for COVID-19, along with research areas, journals, institutions, authors, countries etc. are mined in great depth to obtain useful insights. Performing such analysis leads to some interesting results about the research focused on applications of AI in COVID-19.

Collectively, the main purpose of devising this study is to empirically approach a view of how AI backed research related to COVID-19 has been produced to become impactful. Such a study can be helpful in multiple ways. Major contributions of this article have been compiled below:

1. The research landscape related to AI approaches for COVID-19 is extensively studied.
2. This study provides a quantitative and qualitative analysis of the AI centric scientific research on COVID-19.
3. Different parameters of the recent publications are extracted and studied to explore the landscape of this research area. These are: country-wise analysis, institution-wise analysis, source analysis, productive and influential authors, influential AI subcategories etc.
4. A bibliographic coupling of various parameters and keyword analysis is presented for deeper understanding of the targeted research area.
5. This work provides a platform for future research that can directly build upon the existing literature by referring to the current research trend on AI for COVID-19.
6. The research outcome of the produced scientometric analysis explicitly depicts the overall interest of researchers in the topic of utilizing AI in COVID-19 research.
7. Specifically, Table 3 can be of utmost importance for any new researcher trying to foray into the research of COVID-19, by leveraging the information of the most recent as well as trending work within the area.

Rest of the paper is organized as follows: Section 2 provides a background to the COVID-19 outbreak, while giving a bird’s eye view of the related AI centric research. In Section 3, we discuss the technology utilized to perform the analysis and visualize the data effectively. In the subsequent section, the methodology is laid out followed by the analysis and knowledge mining. The paper is concluded in Section 5.

## Background

Followed by multiple major attacks from viruses such as the severe acute respiratory syndrome coronavirus (SARS-COV), the H1N1 influenza, and the Middle East respiratory syndrome coronavirus (MERS-COV) [15], the world was grappled by the COVID-19 with its first case discovered in late 2019. Growing from this, the virus spread rapidly through geographies. This spread was officially recognized by WHO first [82] on January 21, 2020, followed by declaring it a global health emergency [83]. Finally, through its 40th Situation Report, WHO declared the outbreak as a global pandemic on March 11, 2020 [84, 85].

Fast forward to August 2021, confirmed cases of COVID-19 infected millions across various regions of the globe. This increased motivated researchers from various disciplines to come together to study and develop models that would help in curbing the spread of the deadly virus. This motivation was found to be especially true for the AI research community, due to the evident success of AI within diverse fields. While there were 945 papers published in 2020 with citation count of 3,859, the year 2021 had already seen the publication of 1,491 papers with 11,748 citations. With this, it is evident that interest in the AI centric research on COVID-19 will gradually increase as time progresses, and it is likely to grow over time. Notably, many research works on this topic are already scheduled to be published in the future months.

The study and analysis produced in this paper stands out from all the previous scientometric analyses mentioned in the previous section due to the fact that this work is based on the study of AI-centric research on COVID-19. Apart from this, the scientometric review performed here is extensive in the domain considered, spanning multiple concepts such as trending literature, overall citations, research areas, journals, authors, institutions, countries, keywords and topics, funding agencies, etc. Additionally, an in depth review of the top research articles is performed manually to study the actual use of AI within these works. These articles are then bifurcated into various sub-categories and fields of AI, revealing the interest of researchers, as well as readers in particular contributions of AI. Such a comprehensive study is not visible in any of the existing scientometric analyses presented above.

## Technology utilized

To conduct the investigation on publications related to COVID-19, many technical tools were utilized. We briefly discuss them here.

1. **Web of Science (WoS)**: Maintained by Clarivate Analytics, WoS [24] is a website which hosts multiple databases pertaining to diverse academic disciplines. These databases provide comprehensive data about the citation structure as well as other meta-data about the query presented to the database. In this work, WoS has been queried upon to obtain relevant publication information about COVID-19.
2. **VOSviewer**: It is a software tool [31] utilized for the development of bibliometric networks including multiple entities. These entities can range from being institutions, authors, countries, keywords, journals etc. A comprehensive structure amongst these entities is hence viewed, made possible by VOSviewer. However, there are cases in which the graphs generated by VOSviewer do not have some nodes labeled. Since the overall knowledge gained through such structures is quite good, we ignored the aforementioned drawback and utilized this tool for bibliometric visualizations.
3. **Python**: The rest of the figures in this paper have been drawn with the help of Python 3.6. The bar plots, line plots and frequency histograms have been plotted utilizing the Pandas, Matplotlib, and Numpy modules, as a part of the exploratory data analysis performed.

The details of the scientific publications on the applications of AI for COVID-19 were obtained from the WoS database with the query: TS=(“covid-19”) AND (TS=(“machine learning”) OR TS=(“artificial intelligence”) OR ALL=(“data science”) OR ALL=(“data mining”)). All the major keywords which lie in the framework of AI were included. We considered publications indexed in the Social Science Citation Index (SSCI) as well as the Science Citation Index-Extended (SCI-E) only. This returned a total of 2,434 records of scientific publications when searched on August 15, 2021. These records contain headers such as Title, Authors, Source Title, Publication Date (PD), Citations. Additional information about the records include Research areas, Institutions, Countries and Funding agencies. Each of these headers are analyzed to mine for useful knowledge regarding the ongoing research on COVID-19 with respect to AI. The results are presented below.

## Analysis, results, and discussions

### Citation analysis

Out of the obtained 2,434 scientific publications, each was assigned into slabs depending upon the number of citations. A total of eight such slabs of citations were created: “greater than equal to 0”, “greater than equal to 10”, “greater than equal to 25”, “greater than equal to 50”, “greater than equal to 100”, “greater than equal to 200”, “greater than equal to 400”, and “greater than equal to 500”. On distributing the publications based on their citation counts to these slabs, we see that only one publication obtained more than or equal to 500 citations, which accounts for only 0.04% of the total publications. Furthermore, only one publication so far has garnered less than 500 but more than or equal to 400 citations. The highest percentage share of publications, i.e., 85.54% publications lie in the slab of publications with greater than or equal to 0 citations. This implies that there are 2082 publications out of the total group, which either have citations more than or equal to 1 and less than 10 or remain uncited so far. This implies that due to the recent nature of AI centric publications related to COVID-19, most of the research has not yet garnered citations. This may also be due to the high rate of publications, which makes it difficult for new researchers to cite. The citation structure of all the publications is depicted in Table 1.

**Table 1:** Citation structure of AI centric publications on COVID-19

Slabs of citations	Number of Publications	% share of Publications
>=500	1	0.04
>=400	1	0.04
>=200	5	0.21
>=100	9	0.37
>=50	43	1.77
>=25	68	2.79
>=10	225	9.24
>=0	2082	85.54
Total Publications	2434	100%

### Document types as per WoS database

The research publications can be broadly classified into various types such as articles, corrections, data papers, early access articles, editorial materials, letters, news items, articles in proceedings, and reviews. Based on these categories, the obtained records of publications were classified, as shown in Table 2. It is observed that the highest number of publications (2,021) were classified as articles, followed by 257 and 210 publications classified as reviews and early access, respectively. Apart from these, editorial material, letters, meeting abstract, correction, and data papers consist of 95, 23, 23, 13, and 11 publications, respectively. Two publications each were classified as book chapters, news items, and proceedings papers. Corresponding to this analysis, approximately 83% of the total publications lie in the category of articles, while 210 still lie in the early access. This once again suggests that the research interest on COVID-19, focused on AI, is increasing as time escalates. Hence, more publications have been submitted in recent days.

**Table 2:** AI centric publication on COVID-19 classified into different document types

Document Types	Number of publications	% share of publications*
Article	2021	83.03
Review	257	10.56
Early Access	210	8.63
Editorial Material	95	3.90
Letter	23	0.95
Meeting Abstract	23	0.95
Correction	13	0.53
Data Paper	11	0.45
Book Chapter	2	0.08
News Item	2	0.08
Proceedings Paper	2	0.08

* Percentages in the last column of the table add up to more than 100. This is because one publication may be classified into more than one publication type

### Analysis of top papers on COVID-19

In this section, we analyze the top papers published on COVID-19 with focus on AI. Table 3 depicts the top 50 papers ranked based on total citation (TC) count. The table also depicts the title, authors, journal published in (Source Title), publication month, influential AI subgroups etc. associated with each publication. It can be seen that the top ranked paper, titled “*Prediction models for diagnosis and prognosis of covid-19: systematic review and critical appraisal*” [90] was published in British Medical Journal in April, 2020. This paper gave an overview of the ML prediction models on the early study of COVID-19 which could help in assisting clinical decisions. Recall from Section 4-A that this is the only paper to have garnered more than 500 citations, accounting for 0.04% of the total citations obtained by all the publications considered together. The article titled “*The Impact of COVID-19 Epidemic Declaration on Psychological Consequences: A Study on Active Weibo Users*” [49] was published in International Journal of Environmental Research and Public Health (IJERPH) in March 2020 and ranks second in terms of citations in the top 50 papers list globally with 405 citations. It is noticeable that the top ten papers on the basis of TC have been published within the time period March 2020 – August 2020.

**Table 3:** Top 50 papers published on COVID-19

S.No.	Title	Authors	Source Title	PD	TC	AI subcategories/Review
1	Prediction models for diagnosis and prognosis of covid-19 infection: systematic review and critical appraisal [90]	Wynants, Laure et al.	BMI-British Medical Journal	Apr. 2020	542	Review
2	The Impact of COVID-19 Epidemic Declaration on Psychological Consequences: A Study on Active Weibo Users [49]	Li, Sijia et al.	IJERPH	Mar. 2020	405	Prediction
3	Modified SEIR and AI prediction of the epidemics trend of COVID-19 in China under public health interventions [92]	Yang, Zifeng et al.	Journal of Thoracic Disease	Mar. 2020	339	Prediction
4	Automated detection of COVID-19 cases using deep neural networks with X-ray images [60]	Ozturk, Tulin et al.	Computers in Biology and Medicine	Jun. 2020	330	Classification
5	Non-neuronal expression of SARS-CoV-2 entry genes in the olfactory system suggests mechanisms underlying COVID-19-associated anosmia [12]	Brann, David H. et al.	Science Advances	Jul. 2020	265	Clustering
6	Using Artificial Intelligence to Detect COVID-19 and Community-acquired Pneumonia Based on Pulmonary CT: Evaluation of the Diagnostic Accuracy [48]	Li, Lin et al.	Radiology	Aug. 2020	262	Classification
7	Proteomic and Metabolomic Characterization of COVID-19 Patient Sera [69]	Shen, Bo et al.	Cell	Jul. 2020	254	Classification
8	An interpretable mortality prediction model for COVID-19 patients [91]	Yan, Li et al.	Nature Machine Intelligence	May 2020	191	Classification
9	Artificial intelligence-enabled rapid diagnosis of patients with COVID-19 [56]	Mei, Xueyan et al.	Nature Medicine	Aug. 2020	181	Classification
10	Artificial Intelligence (AI) applications for COVID-19 pandemic [77]	Vaishya, Raju et al.	Diabetes & Metabolic Syndrome-Clinical Research & Reviews	Jul-Aug 2020	142	Review
11	A Comprehensive Review of the COVID-19 Pandemic and the Role of IoT, Drones, AI, Blockchain, and 5G in Managing its Impact [16]	Chamola, Vinay et al.	IEEE Access	May 2020	126	Review
12	Mobility network models of COVID-19 explain inequities and inform reopening [17]	Chang, Serina et al.	Nature	Jan. 2021	124	Data Analysis/Modelling
13	Application of deep learning technique to manage COVID-19 in routine clinical practice using CT images: Results of 10 convolutional neural networks [6]	Ardakani, Ali Abbasian et al.	Computers in Biology and Medicine	Jun 2020	116	Classification
14	Corona Virus (COVID-19) Infodemic and Emerging Issues through a Data Lens: The Case of China [39]	Hua, Jinling and Shaw, Rajib	IJERPH	Apr 2020	116	Data Analysis/Modelling
15	Classification of COVID-19 patients from chest CT images using multi-objective differential evolution-based convolutional neural networks [72]	Singh, Dilbag; Kumar, Vijay; Vaishali; Kaur, Manjit	European Journal of Clinical Microbiology & Infectious Diseases	Jul 2020	114	Classification
16	Towards an Artificial Intelligence Framework for Data-Driven Prediction of Coronavirus Clinical Severity [42]	Jiang, Xiangao et al.	CMC-Computers Materials & Continua	2020	106	Data Analysis/Modelling
17	COVID-19 and artificial intelligence: protecting health-care workers and curbing the spread [55]	McCall, Becky	Lancet Digital Health	Apr 2020	99	Review
18	The COVID-19 pandemic [23]	Ciotti, Marco et al.	Critical Reviews in Clinical Laboratory Sciences	Aug. 2020	98	Review
19	Effects of COVID-19 on hotel marketing and management: a perspective article [41]	Jiang, Yangyang and Wen, Jun	International Journal of Contemporary Hospitality Management	Aug. 2020	97	Review
20	Ranking the effectiveness of worldwide COVID-19 government interventions [38]	Haug, Nils et al.	Nature Human Behaviour	Dec 2020	93	Data Analysis/Modelling
21	Digital technologies in the public-health response to COVID-19 [14]	Budd, Jobie et al.	Nature Medicine	Aug 2020	91	Review
22	Deep Learning COVID-19 Features on CXR Using Limited Training Data Sets [59]	Oh, Yujin et al.	Ieee Transactions on Medical Imaging	Aug 2020	91	Classification and Segmentation
23	Artificial intelligence and machine learning to fight COVID-19 [4]	Alimadadi, Ahmad et al.	Physiological Genomics	Apr 2020	91	Review
24	COVID-19 detection using deep learning models to exploit Social Mimic Optimization and structured chest X-ray images using fuzzy color and stacking [75]	Togacar, Mesut et al.	Computers in Biology And Medicine	Jun 2020	89	Classification
25	Time series forecasting of COVID-19 transmission in Canada using LSTM networks [21]	Chimmula, Vinay Kumar Reddy and Zhang, Lei	Chaos Solitons & Fractals	Jun 2020	88	Forecasting
26	Viral epitope profiling of COVID-19 patients reveals cross-reactivity and correlates of severity [70]	Shrock, Ellen et al.	Science	Nov. 2020	87	Classification
27	In vitro screening of a FDA approved chemical library reveals potential inhibitors of SARS-CoV-2 replication [76]	Touret, Franck et al.	Scientific Reports	Aug. 2020	86	Data Analysis/Modelling
28	Applications of machine learning and artificial intelligence for Covid-19 (SARS-CoV-2) pandemic: A review [46]	Lalmuanawma, Samuel et al.	Chaos Solitons & Fractals	Oct 2020	83	Review
29	Short-term forecasting COVID-19 cumulative confirmed cases: Perspectives for Brazil [65]	Dal Molin Ribeiro, Matheus Henrique et al.	Chaos Solitons & Fractals	Jun 2020	82	Forecasting
30	Machine learning using intrinsic genomic signatures for rapid classification of novel pathogens: COVID-19 case study [63]	Randhawa, G. S. et al.	Plos One	Apr. 2020	82	Classification
31	Artificial Intelligence Augmentation of Radiologist Performance in Distinguishing COVID-19 from Pneumonia of Other Origin at Chest CT [9]	Bai, Harrison X. et al.	Radiology	Sep 2020	81	Classification
32	On the Coronavirus (COVID-19) Outbreak and the Smart City Network: Universal Data Sharing Standards Coupled with Artificial Intelligence (AI) to Benefit Urban Health Monitoring and Management [5]	Allam, Zaheer and Jones, David S.	Healthcare	Mar 2020	80	Review
33	Spatial analysis and GIS in the study of COVID-19. A review [34]	Franch-Pardo, Ivan et al.	Science of The Total Environment	Oct. 2020	78	Review
34	From high-touch to high-tech: COVID-19 drives robotics adoption [94]	Zeng, Zhanjing et al.	Tourism Geographies	May 2020	78	Review
35	CovidGAN: Data Augmentation Using Auxiliary Classifier GAN for Improved Covid-19 Detection [78]	Waheed, Abdul et al.	IEEE Access	May 2020	78	Data Augmentation
36	Within the Lack of Chest COVID-19 X-ray Dataset: A Novel Detection Model Based on GAN and Deep Transfer Learning [51]	Loey, Mohamed et al.	Symmetry-Basel	Apr 2020	74	Classification
37	Predicting COVID-19 Incidence Through Analysis of Google Trends Data in Iran: Data Mining and DL Pilot study [8]	Ayyoubzadeh, M. et al.	Jmir Public Health and Surveillance	Jun 2020	74	Data Analysis/Modelling
38	Impact of Human Disasters and Covid-19 Pandemic on Mental Health: Potential of Digital Psychiatry [26]	Cosic, Kresimir et al.	Psychiatria Danubina	Apr. 2020	74	Review
39	CT quantification of pneumonia lesions in early days predicts progression to severe illness in a cohort of COVID patients [50]	Liu, Fengjun et al.	Theranostics	Apr. 2020	73	Data Analysis/Modelling
40	Diagnostic methods and potential portable biosensors for coronavirus disease 2019 [27]	Cui, Feiyun and Zhou, H. Susan	Biosensors & Bioelectronics	Oct. 2020	72	Review
41	Can AI Help in Screening Viral and COVID-19 Pneumonia? [22]	Chowdhury, Muhammad E. H. et al.	IEEE Access	July 2020	71	Classification
42	A deep learning algorithm using CT images to screen for Corona virus disease (COVID-19) [81]	Wang, Shuai et al.	European Radiology	Aug 2021	70	Classification
43	AI-Driven Tools for Coronavirus Outbreak: Need of Active Learning and Cross-Population Train/Test Models on Multitudinal/Multimodal Data [68]	Santosh, K. C.	Journal of Medical Systems	Mar. 2020	70	Review
44	A Weakly-Supervised Framework for COVID-19 Classification and Lesion Localization From Chest CT [79]	Wang, Xinggang et al.	IEEE Transactions on Medical Imaging	Aug 2020	68	Classification
45	Impact of COVID-19 pandemic on information management research and practice: Transforming education, work and life [30]	Dwivedi, Y. K. et al.	Int. Journal of Information Management	Dec 2020	67	Review
46	Explainable Deep Learning for Pulmonary Disease and Coronavirus COVID-19 Detection from X-rays [13]	Brunese, Luca et al.	Computer Methods and Programs In Biomedicine	Nov 2020	65	Classification
47	Psychological stress of medical staffs during outbreak of COVID-19 and adjustment strategy [88]	Wu, Wenzhi et al.	Journal of Medical Virology	Oct 2020	62	Data Analysis/Modelling
48	Mechanism of baricitinib supports artificial intelligence-predicted testing inCOVID-19 patients [73]	Stebbing, Justin et al.	Embo Molecular Medicine	Aug. 2020	60	Data Analysis/Modelling
49	Mutations Strengthened SARS-CoV-2 Infectivity [19]	Chen, Jiahui et al.	Journal of Molecular Biology	Sep. 2020	59	Data Analysis/Modelling
50	Identification of COVID-19 can be quicker through artificial intelligence framework using a mobile phone-based survey when cities and towns are under quarantine [64]	Srinivasa Rao, Arni S. R. and Vazquez, Jose A.	Infection Control and Hospital Epidemiology	Jul. 2020	59	Classification

**Fig. 2:**
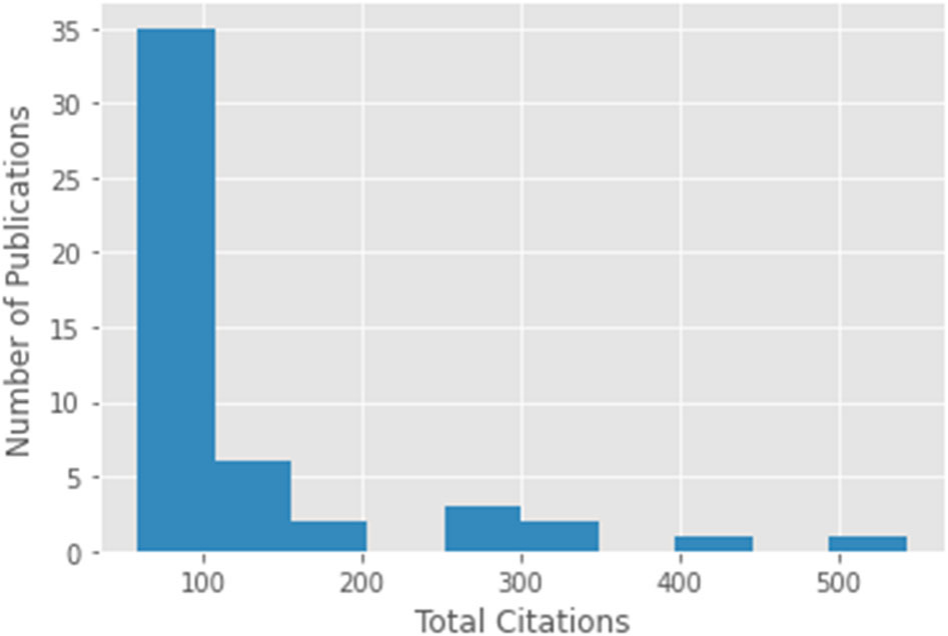
Number of publications vs. total citations histogram for top-50 papers

**Fig. 3:**
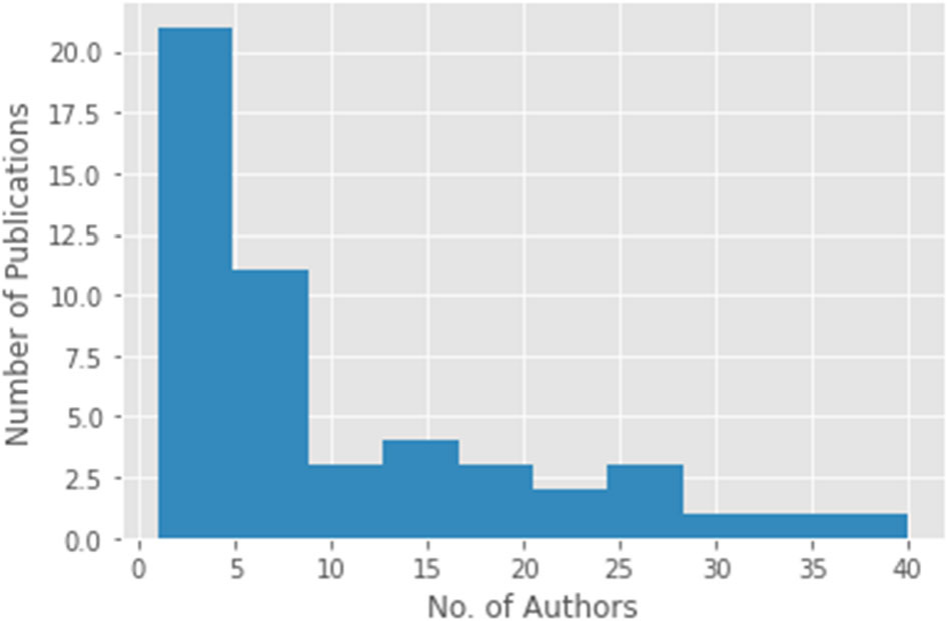
Number of authors and frequency of publications, histogram for top-50 papers

Another analysis of TC for top 50 papers reveals interesting findings. In Fig. 2, a frequency histogram of publications according to TC is depicted. To obtain this figure, the entire range of TC was divided into 10 bins. Each bar against the bin depicts the number of publications falling in that bin (having obtained corresponding number of citations). Analyzing this frequency histogram demonstrates that most publications in the top 50 list have garnered citations between 60–100. The number of publications decrease as number of citations increase in the bins. Figure 3 is another frequency distribution histogram depicting the relationship of the number of authors with the impact of the publication. In this figure, the X-axis denotes the bins of number of authors, while the bars depict the number of papers in that bin. We decided upon the same 10 bins for this analysis. The highest number of publications in the top 50 list have 2 to 9 authors. The number of publications decreases as the number of authors within each publication increases. It is very interesting to note that there is one paper in the top 50 papers list where the number of authors is 40, which is indicative of an interdisciplinary research work.

### Technical analysis of top articles based on AI centric COVID-19 research

Due to the significant applicative capabilities of AI, it is crucial to dive deep into its impact on the research of COVID-19. In this section, specifics of different fields and types of AI are discussed, with respect to the top-50 research articles provided in Table 3. To begin with the topmost highly cited research article, ‘*Prediction models for diagnosis and prognosis of COVID-19: Systematic review and critical appraisal*’, [90] studied published articles and preprints of prediction models of COVID-19, meant for diagnosis, prognosis as well as detection of people in general population with increased risk of infection. In this article, AI, specifically through a text analysis tool, was utilized to prioritize research materials relevant to the study. In the next article from this list, [49], posts from Weibo, a Chinese micro-blogging website were considered to study the psychological impacts of COVID-19 on the former’s users. This work used the Online Ecological Recognition (EOR), based on the broad category of ML predictive models. The article titled ‘*Modified SEIR and AI prediction of the epidemics trend of COVID-19 in China under public health interventions*’, [92] ranked third highest in terms of citations, developed a simple neural network structure based on long-short term memory (LSTM) which predicted peak of infection in mainland China. Automatic detection of COVID-19 through binary and multi-class classifications was performed in [60]. The article [12], with the fifth-highest citations, employed AI as a tool for primary filtration of gene data through doublet cell detection. Subsequently, a classification deep neural net called the COVNet was proposed in [48], to identify COVID-19 and community-acquired pneumonia infections from no infections using volumetric chest CT scans. The article ‘*Proteomic and Metabolomic Characterization of COVID-19 Patient Sera*’ [69] presented an ML-based random forest classifier to identify patients with severe COVID-19 infections, following their protein and metabolite characteristics. With interpretability as a highlighting concern for AI models, authors of [91] developed a discriminative ML model for the classification of the most important biomarkers of patient mortality, concerning the COVID-19 disease. The XGBoost model was considered for this task. In [56], the authors studied CT scans for presented patients using convolutional neural networks to learn specific characteristics. Since these scans presented normal radiological findings in early stages of infection multilayer perceptron (MLP) classifiers were put to use for classification of patients using more clinical information. The article with tenth highest number of citations [77], reviewed research articles to identify most significant seven AI applications pertaining to detection of COVID-19.

In the subsequent data, multiple review articles [4, 14, 16, 23, 41, 55] studied either the publication landscape, or the impact on COVID-19 responses due to AI and/or other digital technologies such as blockchain, IOT etc. Furthermore, a total of four [6, 59, 72, 75] articles within the top 10–25 articles with highest citations, dealt with the detection of COVID-19 in patients through segmentation and classification on chest X-Ray images. It is interesting to note that most of such models are deep learning models, hence indicating the prowess of this AI trajectory in the detection of COVID-19. To top these off, four [17, 38, 39, 42] articles in the top 10–25 presented data analysis and modelling results and frameworks, few of which dealt with big data analysis, susceptible-exposed-infected-recovered (SEIR) models and mobility networks. Apart from these, only one LSTM based forecasting model for COVID-19 [21] managed to rank 25th in garnering citations.

In summary for the rest of the articles ranked from 26 to 50 for top citations, highest number of articles (nine) proposed or dealt with classification models [9, 13, 22, 51, 63, 64, 70, 79, 81]. This was followed by eight review articles [5, 26, 27, 30, 34, 46, 68, 94], and six articles on data analysis and data modelling frameworks [8, 19, 50, 73, 76, 88]. There is only one paper however, which presented data augmentation methods to aid in the task of COVID-19 detection [78].

From the above analysis, it is easy to observe that research on classification models for detecting the presence and/or absence of COVID-19 in patients is quite prominent and also garners the most interest. Amongst these, DL models reign due to their highly accurate results, given a good amount of data. A review of the impact of AI-centric research on COVID-19 is a second favorite amongst researchers to study and present the effect that COVID-19 has had on the world in different ways. Data analysis and various methods of modelling the same have also grasped the interest of researchers to imply essential results through the study of data obtained from multitudes of resources and in diverse modalities.

### Research areas of publications

In Table 4, we list the top 50 research areas of publications obtained from WoS. The top 5 research areas include “Computer Science”, “Engineering”, “Science Technology Other Topics”, “Medical Informatics”, and “Health Care Sciences Services” with 547, 334, 230, 210, and 201 number of publications in each research area, respectively. It can be seen clearly that only the top 5 research areas have more than 200 publication records, collectively accounting for more than 62% share of total publications (TP). The highest % share, i.e. 22.47% of TP is in the research area “Computer Science”, which is approximately 11% more than the % share of the “Engineering” research area.

**Table 4:** Research areas of AI centric research publications on COVID-19

S. No.	Research Areas	Publication Records	% share of TP
1	Computer Science	547	22.47
2	Engineering	334	13.72
3	Science Technology Other Topics	230	9.45
4	Medical Informatics	210	8.63
5	Health Care Sciences Services	201	8.26
6	General Internal Medicine	171	7.03
7	Environmental Sciences Ecology	142	5.83
8	Public Environmental Occupational Health	139	5.71
9	Telecommunications	120	4.93
10	Physics	99	4.07
11	Chemistry	98	4.03
12	Radiology Nuclear Medicine Medical Imaging	97	3.99
13	Materials Science	96	3.94
14	Biochemistry Molecular Biology	90	3.70
15	Business Economics	84	3.45
16	Mathematics	83	3.41
17	Mathematical Computational Biology	73	3.00
18	Pharmacology Pharmacy	68	2.79
19	Research Experimental Medicine	60	2.47
20	Biotechnology Applied Microbiology	49	2.01
21	Psychology	44	1.81
22	Life Sciences Biomedicine Other Topics	43	1.77
23	Information Science Library Science	36	1.48
24	Infectious Diseases	35	1.44
25	Education Educational Research	33	1.36
26	Instruments Instrumentation	33	1.36
27	Neurosciences Neurology	30	1.23
28	Social Sciences Other Topics	27	1.11
29	Immunology	26	1.07
30	Psychiatry	26	1.07
31	Cell Biology	25	1.03
32	Cardiovascular System Cardiology	24	0.99
33	Energy Fuels	23	0.95
34	Respiratory System	22	0.90
35	Genetics Heredity	21	0.86
36	Oncology	21	0.86
37	Imaging Science Photographic Technology	20	0.82
38	Automation Control Systems	19	0.78
39	Endocrinology Metabolism	17	0.70
40	Microbiology	17	0.70
41	Operations Research Management Science	17	0.70
42	Biophysics	15	0.62
43	Government Law	14	0.58
44	Surgery	14	0.58
45	Virology	14	0.58
46	Meteorology Atmospheric Sciences	13	0.53
47	Remote Sensing	13	0.53
48	Geology	11	0.45
49	Mathematical Methods In Social Sciences	11	0.45
50	Communication	10	0.41

While one might think that research related to COVID-19 focused even on AI is being published only in medical related journals, this is not the case in fact. The list of top 50 research areas indicates that AI centric research on COVID-19 has drawn researchers from diverse fields, viz. science and technology, engineering, chemistry, social science, psychology etc. The minimum number of publications in top 50 is atleast 10.

### Analysis of journals

The destination journal of a publication is one of the most important factors while analyzing any publication data. Journals with high TP are the most productive journals, whereas the ones with the highest TC are the most influential journals. Tables 5 and 6, respectively, list the top 20 most productive and most influential journals with AI centric publications related COVID-19.

**Table 5:** Top 20 most productive journals

S. No.	Journal	TP	TC	CPP
1	Journal of Medical Internet Research	90	429	4.77
2	IEEE Access	84	647	7.70
3	International Journal of Environmental Research and Public Health	53	765	14.43
4	Scientific Reports	52	185	3.56
5	CMC-Computers Materials & Continua	44	171	3.89
6	Plos One	44	309	7.02
7	Sustainability	28	116	4.14
8	Applied Sciences-Basel	26	68	2.62
9	Chaos Solitons & Fractals	25	560	22.40
10	Computers in Biology and Medicine	19	588	30.95
11	JMIR Medical Informatics	19	62	3.26
12	Diagnostics	18	79	4.39
13	Sensors	16	40	2.50
14	Healthcare	15	88	5.87
15	Applied Intelligence	14	100	7.14
16	Applied Soft Computing	14	177	12.64
17	International Journal of Advanced Computer Science and Applications	14	5	0.36
18	Journal of Clinical Medicine	14	96	6.86
19	Lancet Digital Health	13	232	17.85
20	Proceedings of the National Academy of Sciences of the United States of America	13	72	5.54

**Table 6:** Top 20 most influential journals

S. No.	Journal	TC	TP	CPP
1	International Journal of Environmental Research and Public Health	765	53	14.43
2	IEEE Access	647	84	7.70
3	Computers in Biology and Medicine	588	19	30.95
4	Chaos Solitons & Fractals	560	25	22.40
5	Radiology	451	11	41.00
6	Journal of Medical Internet Research	429	90	4.77
7	Plos One	309	44	7.02
8	IEEE Transactions on Medical Imaging	264	5	52.80
9	Diabetes & Metabolic Syndrome-Clinical Research & Reviews	251	10	25.10
10	Nature Machine Intelligence	244	9	27.11
11	Lancet Digital Health	232	13	17.85
12	Scientific Reports	185	52	3.56
13	Applied Soft Computing	177	14	12.64
14	Cmc-Computers Materials & Continua	171	44	3.89
15	Journal Of Medical Systems	151	7	21.57
16	Science of The Total Environment	146	7	20.86
17	Nature Communications	130	9	14.44
18	International Journal of Information Management	127	5	25.40
19	European Radiology	124	12	10.33
20	Sustainability	116	28	4.14

The “Journal of Medical Internet Research” is the most productive journal with 90 publications, followed by “IEEE Access” with 84 publications. Other journals among the top 5 ranks are “International Journal of Environmental Research and Public Health”, “Scientific Reports”, and “Computers Materials & Continua” possessing 53, 52 and 44 publications, respectively. The journals in Table 5 consists of a mix of domains from science, medical and engineering. Qualitatively, though the journals may appear belonging to several domains, the scope of each of these journals targets the applicability of AI and ML approaches.

The two journals with highest citations per paper (CPP) in the list are “Computers in Biology and Medicine” (CIBM) and “Chaos Solitons & Fractals” (CSF) with CPP of 30.95 and 22.40. Both journals publish work pertaining to computational biology in common, and thus, they support the publications on COVID-19, which addressed AI, ML, and data analytics. Despite publishing only 19 and 25 papers, these received decent citations of 588 and 560, respectively.

Furthermore, the highly cited papers from CIBM targeted the use of DL methods for the intrinsic analysis on COVID-19. These papers were: “*Automated detection of COVID-19 cases using deep neural networks with X-ray images*”, “*Application of deep learning technique to manage COVID-19 in routine clinical practice using CT images: Results of 10 convolutional neural networks*”, and “*COVID-19 detection using deep learning models to exploit Social Mimic Optimization and structured chest X-ray images using fuzzy color and stacking approaches*” etc. The highly cited papers from CSF targeted time series forecasting of COVID-19 and some thorough review on the Applications of machine learning and artificial intelligence for COVID-19.

In Table 6 the top 20 highly influential journals are listed, which garnered the most attention from other publications. The top two journals are “International Journal of Environmental Research and Public Health” (TC = 765) and “IEEE Access” (TC = 647). CIBM and CSF, both journals with the highest CPP above, rank third and fourth in the list of most influential journals. The top seven journals in this list account for more than 300 citations which, apart from the top four, includes “Radiology”, “Journal of Medical Internet Research”, and “PLOS One” with TC of 451, 429, and 309, respectively.

The highest CPP of 52.80 is received by “IEEE Transactions on Medical Imaging” which targets the application of AI and ML in the medical image processing. It is followed by “Radiology” and CIBM with CPP of 41 and 30.95, respectively. It is observed that there are many open access journals in both the lists as it was the need of the hour to publish articles as soon as possible and within the reach of every academician and scientist. It also points out the extensive amount of funding invested for such research. The funding details are discussed at length in Section 4.12.

**Fig. 4:**
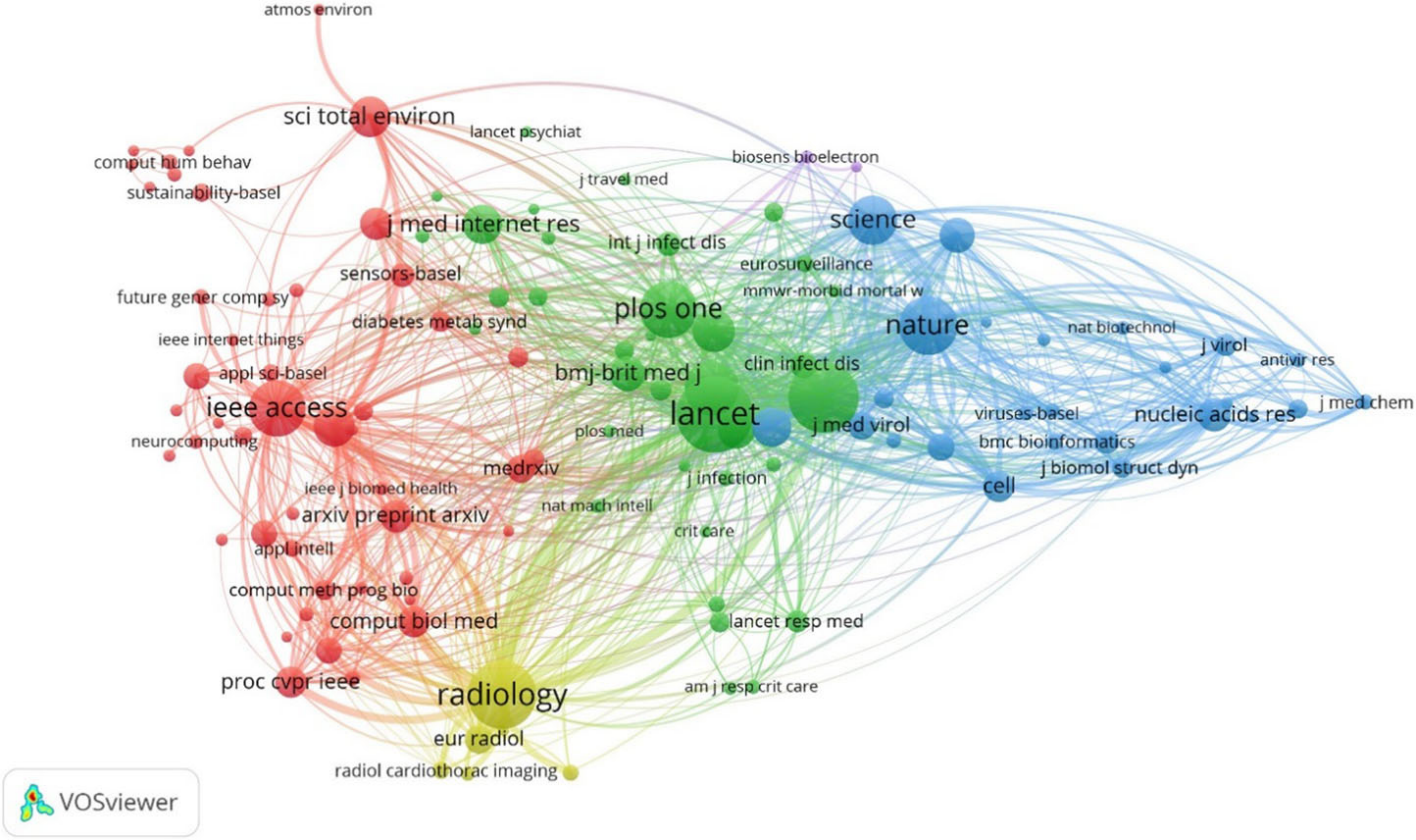
Co-citation analysis between journals

**Fig. 5:**
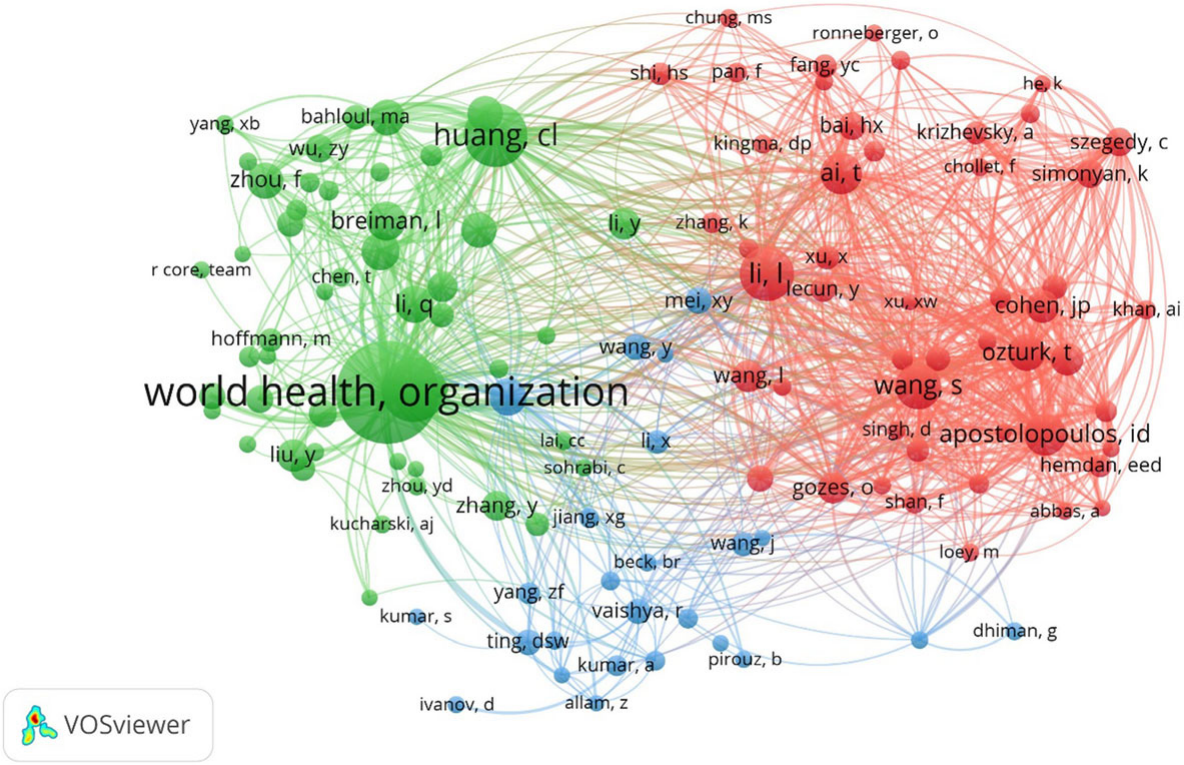
Co-citation amongst authors

Figure 4 depicts the co-citation mapping between the journals that have published AI centric papers related to COVID-19. Since there were 30,911 sources, we considered only those journals which have received at least 100 citations. This resulted in a total of 116 journals. Co-citation analysis for a journal considers the total number of citations and compute how many times a journal paper has been cited in another journal. From Fig. 5, it is observed that “IEEE Access”, “Radiology”, “Lancet”, “PLOS One”, and “Nature” are the largest nodes indicating that they have been cited more by the other journals for COVID-19 research related to AI. The clusters distinguished by different colors indicate more citations between them. For example, “Arxiv preprint” has articles which have cited more papers from IEEE access, hence they are in same cluster with strong link strength.

### Analysis of authors

Akin to journals, the top authors of publications related to COVID-19 focused on AI are categorized as the top 20 most productive authors in Table 7. The difference in the number of publications among these top 20 authors is not much significant. Al-Turjman tops this list with a TP of 9, followed by Duong and Hassanien with a TP of 8 and 7, respectively. Then there are 8 authors with 6 publications each. Rest of the authors in the list have 5 papers. Interestingly, Acharya has the highest CPP of 92.40 with only 5 papers. On the other hand, Ittamalla with 6 papers has the lowest CPP of 0.50. This is because Acharya’s publication has targeted AI and medical informatics which were the centre of attraction among researchers during pandemic.

**Table 7:** Top 20 most productive authors

S. No.	Author	TP	TC	CPP
1	Al-Turjman, Fadi	9	115	12.78
2	Duong, Tim Q.	8	90	11.25
3	Hassanien, Aboul Ella	7	91	13.00
4	Ittamalla, Rajesh	6	3	0.50
5	Kuhl, Ellen	6	42	7.00
6	Li, Haifang	6	78	13.00
7	Mahmud, Mufti	6	30	5.00
8	Mukherjee, Bhramar	6	25	4.17
9	Xue, Jia	6	466	77.67
10	Zhang, Wei	6	56	9.33
11	Zhu, Tingshao	6	456	76.00
12	Acharya, U. Rajendra	5	462	92.40
13	Javaid, Mohd	5	188	37.60
14	Abdulkareem, Karrar Hameed	5	103	20.60
15	Wang, Rui	5	85	17.00
16	Magazzino, Cosimo	5	84	16.80
17	Mele, Marco	5	84	16.80
18	Pirouz, Behrouz	5	76	15.20
19	Linka, Kevin	5	35	7.00
20	Frenkel-Morgenstern, Milana	5	29	5.80

Table 8 shows the list of top 20 most influential authors, i.e., authors ranked based on their TC. First rank is obtained by Xue with a TC of 466 with only 6 papers. Other than Xue, there are three other authors with more than 400 citations. These authors are Acharya (TC = 462), Zhu (TC = 456), and Li (TC = 424). Then there are 10 authors with more than 100 citations. All the authors in this list have published atleast 3 papers. Another very interesting observation that can be made here is that, if we compare Tables 7 and 8, we find that only 8 authors from the most productive list find their place in the most influential authors. Also, the 3 topmost influential authors also fall in the list of top 20 most productive authors, which indicates that their quality work is well received by the research community.

**Table 8:** Top 20 most influential authors

S. No.	Author	TC	TP	CPP
1	Xue, Jia	466	6	77.67
2	Acharya, U. Rajendra	462	5	92.40
3	Zhu, Tingshao	456	6	76.00
4	Li, Sijia	424	4	106.00
5	Javaid, Mohd	188	5	37.60
6	Haleem, Abid	186	4	46.50
7	Vaishya, Raju	144	3	48.00
8	Kaur, Manjit	126	3	42.00
9	Kumar, Vijay	126	3	42.00
10	Singh, Dilbag	126	3	42.00
11	Khalifa, Nour Eldeen M.	121	4	30.25
12	Loey, Mohamed	121	4	30.25
13	Al-Turjman, Fadi	115	9	12.78
14	Abdulkareem, Karrar Hameed	103	5	20.60
15	Zheng, Chuansheng	97	3	32.33
16	Alamoodi, A. H.	92	3	30.67
17	Albahri, A. S.	92	3	30.67
18	Zaidan, A. A.	92	3	30.67
19	Zaidan, B. B.	92	3	30.67
20	Hassanien, Aboul Ella	91	7	13.00

The co-citation amongst authors describes the citing relationship between two authors i.e. the number of times an author is cited by the others. The connections resulting from a co-citation versus authors mapping is presented in Fig. 5. For better visualization, the minimum number of citations of each author has been kept at 50 which among 57,770, resulted in only 125 authors. The node corresponding to the WHO is the largest indicating that the articles published by them are cited the most by all the authors, which is very relevant and justified. Authors in the same color cluster indicate the strong co-citation among them. Li and Wang gathered much attention with respect to the red color clusters. Similarly, Huang is another author from green cluster who has been cited most.

### Analysis of institutions

Institutions play a key role in the promotion of scientific research. In the case of AI centric research on COVID-19, the top 20 most productive institutions are given in Table 9. It can be observed that the maximum number of publications are from researchers from the Harvard Medical School with a TP of 48, while Huazhong University of Science and Technology comes second in this list with a TP of 41. Only these two institutions have publications over 40, as rest of them have at most 30 publications. Other three institutions i.e., Chinese Academy of Sciences, Stanford University, and King Saud University have 30 publications each, hence, they are ranked on the basis of high TC. Notably, two topmost institutions based on CPP are University of Toronto (CPP = 27.32), and University of Oxford (CPP = 24.22). This indicator establishes the quality-oriented publications from these institutions.

**Table 9:** Top 20 most productive institutions

S. No.	Institutions	TP	TC	CPP
1	Harvard Med Sch	48	495	10.31
2	Huazhong Univ Sci & Technol	41	650	15.85
3	Chinese Acad Sci	30	601	20.03
4	Stanford Univ	30	233	7.77
5	King Saud Univ	30	115	3.83
6	Univ Oxford	27	654	24.22
7	Imperial Coll London	24	175	7.29
8	Univ Chinese Acad Sci	23	514	22.35
9	Shanghai Jiao Tong Univ	24	139	5.79
10	King Abdulaziz Univ	23	126	5.48
11	Univ Toronto	22	601	27.32
12	Natl Univ Singapore	22	135	6.14
13	Univ Michigan	22	117	5.32
14	Taif Univ	23	67	2.91
15	Massachusetts Gen Hosp	21	399	19.00
16	MIT	21	163	7.76
17	Icahn Sch Med Mt Sinai	19	366	19.26
18	Zhejiang Univ	20	289	14.45
19	Fudan Univ	19	203	10.68
20	Cairo Univ	18	238	13.22

This also makes University of Oxford as the most influential institution with the highest TC of 654, while Huazhong University of Science and Technology has the second highest TC amounting to 650, closely trailed by Medical University of Vienna with TC of 649. This can be viewed from Table 10, where the top 20 most influential institutions for AI centric research on COVID-19 are given. There are total of 12 institutions with more than 500 citations which is a great achievement considering almost 1 year of publication. The highest CPP of 129.80 is attained by Medical University of Vienna, which is remarkable to achieve through just 5 publication within few months. Even the lowest citation count in this list is 366, obtained by Icahn School of Medicine at Mount Sinai, emphasizing that there is a huge number of works that are being published in AI centric research on COVID-19.

**Table 10:** Top 20 most influential institutions

S. No.	Institutions	TC	TP	CPP
1	Univ Oxford	654	27	24.22
2	Huazhong Univ Sci & Technol	650	41	15.85
3	Med Univ Vienna	649	5	129.80
4	Maastricht Univ	628	7	89.71
5	Chinese Acad Sci	601	30	20.03
6	Univ Toronto	601	22	27.32
7	Charite Univ Med Berlin	574	10	57.40
8	Berlin Inst Hlth	573	9	63.67
9	Humboldt Univ	566	9	62.89
10	Free Univ Berlin	559	7	79.86
11	Univ Chinese Acad Sci	514	23	22.35
12	Asia Univ	511	11	46.45
13	Harvard Med Sch	495	48	10.31
14	Ngee Ann Polytech	462	5	92.40
15	Nankai Univ	418	7	59.71
16	Massachusetts Gen Hosp	399	21	19.00
17	Univ Hong Kong	385	17	22.65
18	Firat Univ	378	8	47.25
19	Minist Hlth	370	5	74.00
20	Icahn Sch Med Mt Sinai	366	19	19.26

### Analysis of countries

The first case of COVID-19 was reported in China in December, 2019. Since then, strict isolation measures were put into practice to prevent the spread of this disease. However, reports on people being infected by COVID-19 were soon registered in various countries such as Thailand, Korea, Japan etc. [80]. Worldometer [87] listed 222 countries/regions as being affected by COVID-19. Due to this, the study of interest amongst researchers from various geographies becomes crucial. The total number of countries dealing with AI centric research on COVID-19 returned from our query was 110. It shows that around 50% of the affected countries started working with the applications of AI and ML in the field of COVID-19.

For ease of analysis, these countries were divided into two lists of top 20 most productive and most influential countries, as shown in Tables 11 and 12, respectively. Referring to both these tables, it can be observed that USA, China, United Kingdom, and India retain the top 4 ranks. The order in which these countries are ranked based on their TP is USA, China, India, and United Kingdom with a TP count of 721, 326, 344 and 236, respectively. Notably, second ranked China has less than around 50% of total publications than USA. In terms of TC, USA, USA, England, and India obtain counts of 4750, 4130, 2787 and 1803, respectively. The highest CPP of 47.75% is obtained by Belgium with TC of 955 in just 20 publications. Furthermore, we also observe that Asia as a continent has the most number of productive and influential countries in the top 20 list. Specifically, six countries out of the 20 most productive countries lie in Asia. The second spot is taken up by USA.

**Table 11:** Top 20 most productive countries in the world

S. No.	Country	TP	TC	CPP
1	USA	721	4750	6.59
2	Peoples R China	386	4130	10.70
3	India	344	1803	5.24
4	United Kingdom	236	2787	11.81
5	Italy	176	1801	10.23
6	Saudi Arabia	156	518	3.32
7	Spain	130	493	3.79
8	Canada	128	1331	10.40
9	Australia	113	772	6.83
10	Germany	89	987	11.09
11	South Korea	77	463	6.01
12	Turkey	77	745	9.68
13	Pakistan	74	274	3.70
14	France	71	452	6.37
15	Egypt	68	454	6.68
16	Switzerland	59	211	3.58
17	Brazil	58	338	5.83
18	Iran	58	480	8.28
19	Malaysia	49	478	9.76
20	Singapore	49	699	14.27

**Table 12:** Top 20 most influential countries in the world

S. No.	Country	TC	TP	CPP
1	USA	4750	721	6.59
2	Peoples R China	4130	386	10.70
3	United Kingdom	2787	236	11.81
4	India	1803	344	5.24
5	Italy	1801	176	10.23
6	Canada	1331	128	10.40
7	Germany	987	89	11.09
8	Belgium	955	20	47.75
9	Netherlands	788	36	21.89
10	Australia	772	113	6.83
11	Turkey	745	77	9.68
12	Singapore	699	49	14.27
13	Austria	695	26	26.73
14	Taiwan	624	40	15.60
15	Japan	589	31	19.00
16	Saudi Arabia	518	156	3.32
17	Spain	493	130	3.79
18	Iran	480	58	8.28
19	Malaysia	478	49	9.76
20	South Korea	463	77	6.01

### Bibliographic coupling amongst various data

Bibliographic coupling is a measure that indicates the possibility of similarity between works done by two entities [53]. It is intuitively defined as the number of times more than one entity cites one common entity. These entities may be institutions, countries, authors etc. We have considered all such different entities and obtained results for each of them.

#### Bibliographic coupling amongst authors

**Fig. 6:**
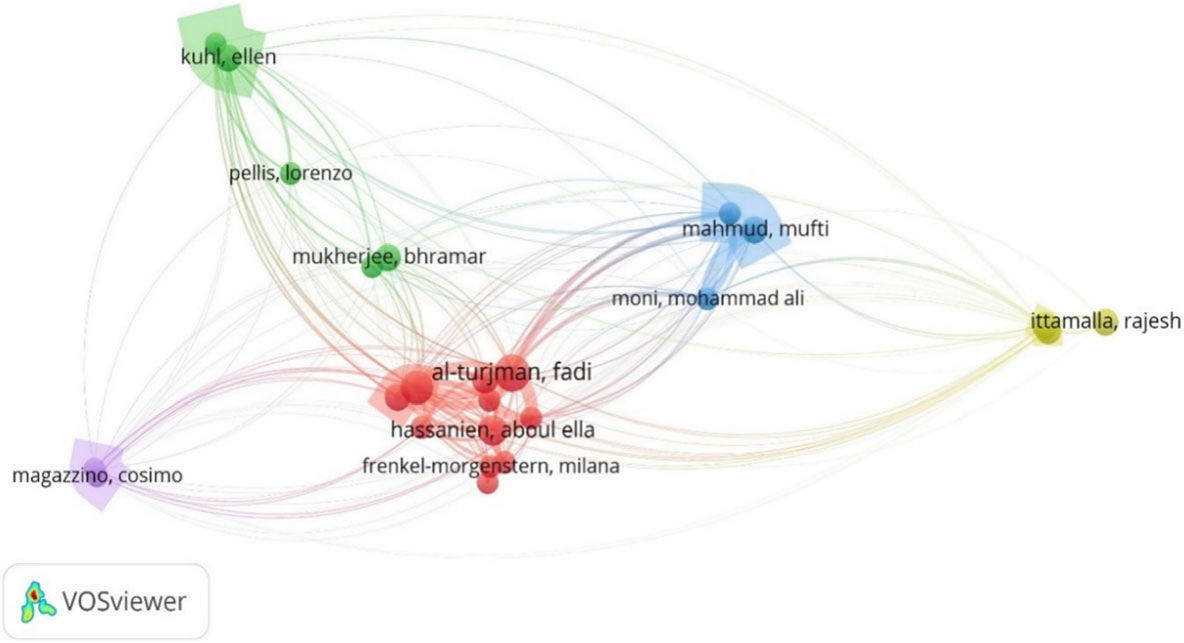
Bibliographic coupling amongst authors

Figure 6 shows the bibliographic relationship amongst different authors of AI centric publications related to COVID-19. The different colors represent different clusters within the network. The size of the bubbles indicates those authors, publications by whom are the most cited in their cluster. For example, publications by Kuhl have been cited highly when compared to other authors in the green cluster. Similarly, Mahmud (blue cluster), Magazzino (voilet cluster), Ittamalla (yellow cluster) and Al-turjman (red cluster) are the main players in their respective clusters. Each cluster is based on of high frequency citations amongst the authors within that cluster. Furthermore, the curves linking the nodes in the clusters have different width. Higher width of the curves demonstrates more matches in the references of the publications by the two authors.

#### Bibliographic coupling amongst countries

**Fig. 7:**
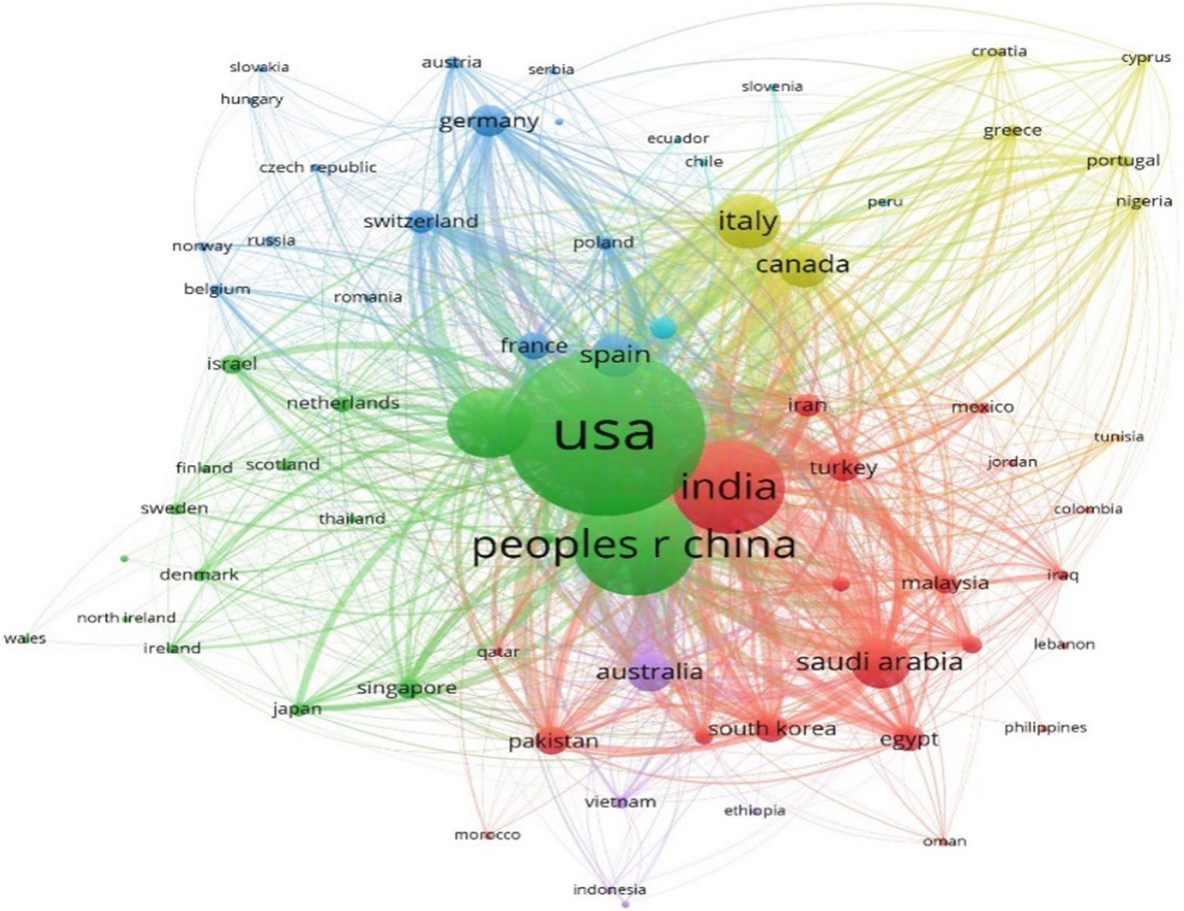
Bibliographic coupling amongst countries

The bibliographic coupling network amongst the countries depicts the similarities in references between publications by two countries, as shown in Fig. 7. It can be clearly observed that the main players in this network are USA and China (green cluster), Canada and Italy (yellow cluster), India and Saudi Arabia (red cluster), Germany (blue cluster), Australia (violet cluster) etc. Curves connecting nodes corresponding to Switzerland and Spain, USA and Canada, India and Turkey etc. possess the most width indicating the similarity in the references of their works.

#### Bibliographic coupling amongst institutions

**Fig. 8:**
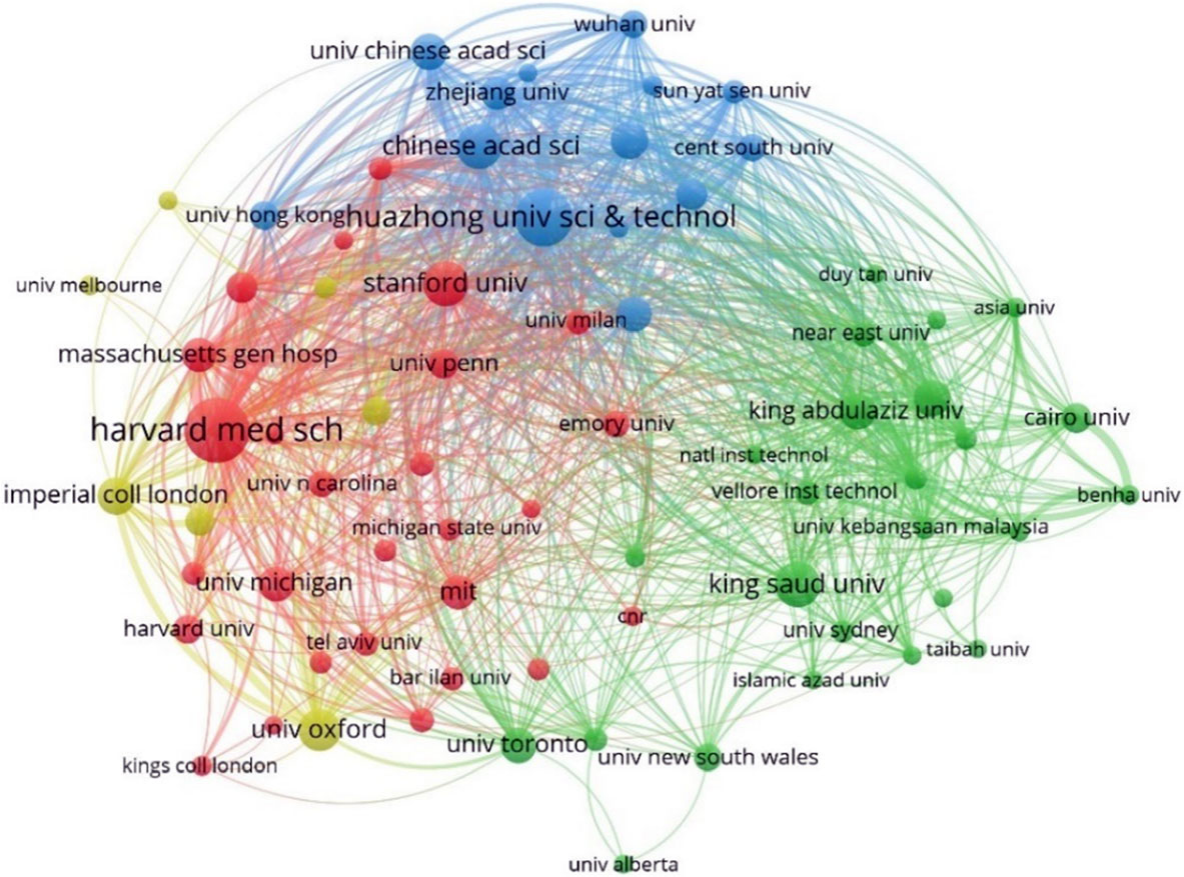
Bibliographic coupling amongst institutions

A dense network of bibliographic relationships between institutions worldwide is shown in Fig. 8. Huazhong University of Science and Technology (blue cluster), Harvard Medical School (red cluster), and King Saud University, University of Toronto, and King Abdulaziz University (green cluster) can be identified as the main institutions in each cluster within the network that receive high citations within their respective clusters. The high density of connections indicates that the citation network amongst institutions is extremely diverse.

#### Bibliographic coupling amongst journals

**Fig. 9:**
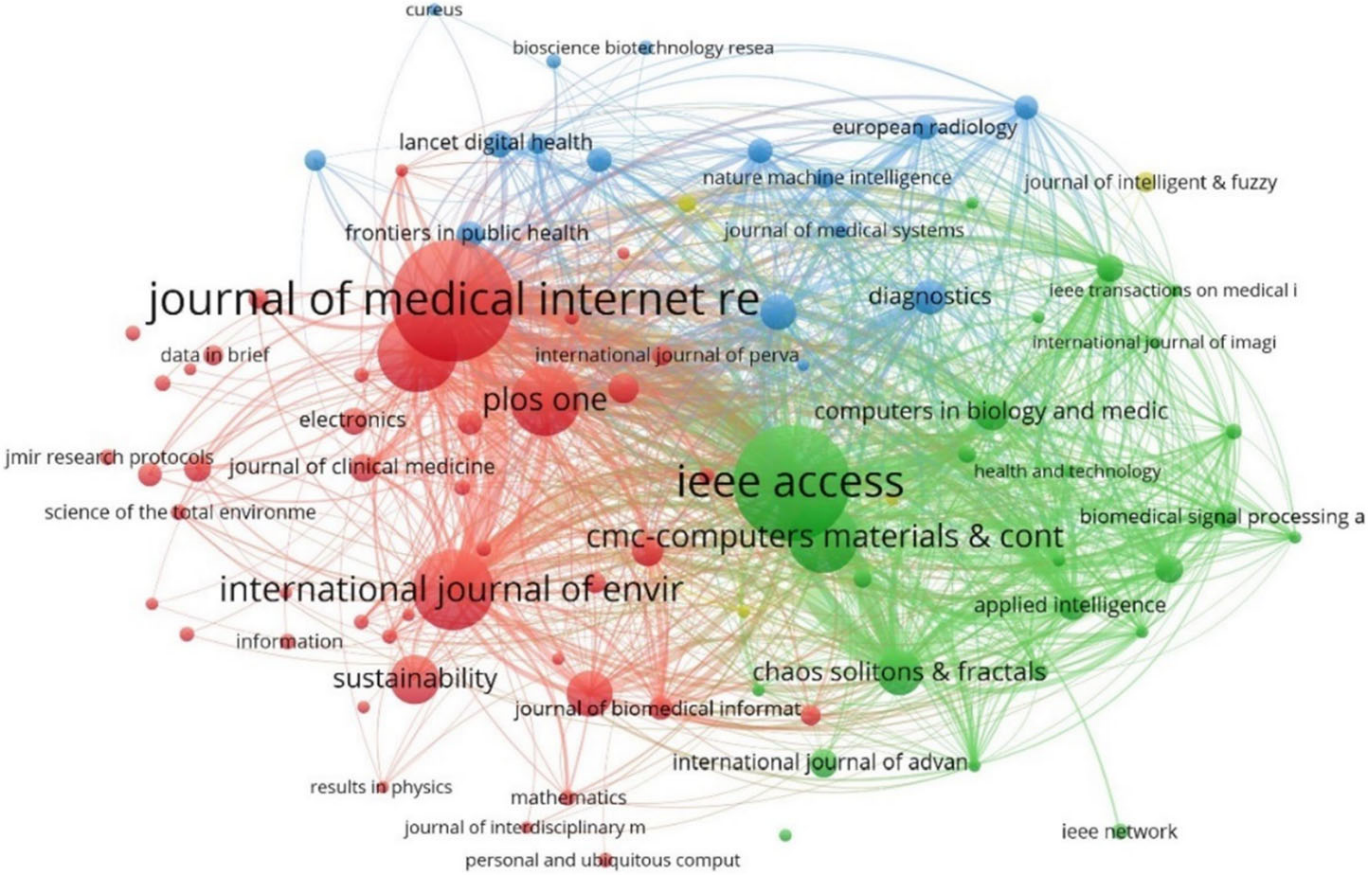
Bibliographic coupling amongst journals

Various clusters can be observed from the bibliographic coupling network between journals as shown in Fig. 9. Amongst these the “Journal of Medical Internet Research” and “PLOS One” (red cluster), “IEEE Access” (green cluster), “Diagnostics” (blue cluster), “Journal of Intelligent & Fuzzy Systems” (yellow cluster) etc. are the main entities in their respective clusters of the entire network. Journals such as “IEEE Access”, “Journal of Medical Internet Research”, “International Journal of Environmental Research and Public Health”, and “ Materials & Continua” have been more frequently cited by the other journals within their respective clusters. It is worth noticing that the journals that do not appear in any clusters indicates that these journals, being important journals within their respective clusters, do not share any common references with any other journal in the network.

### Keyword analysis

**Fig. 10:**
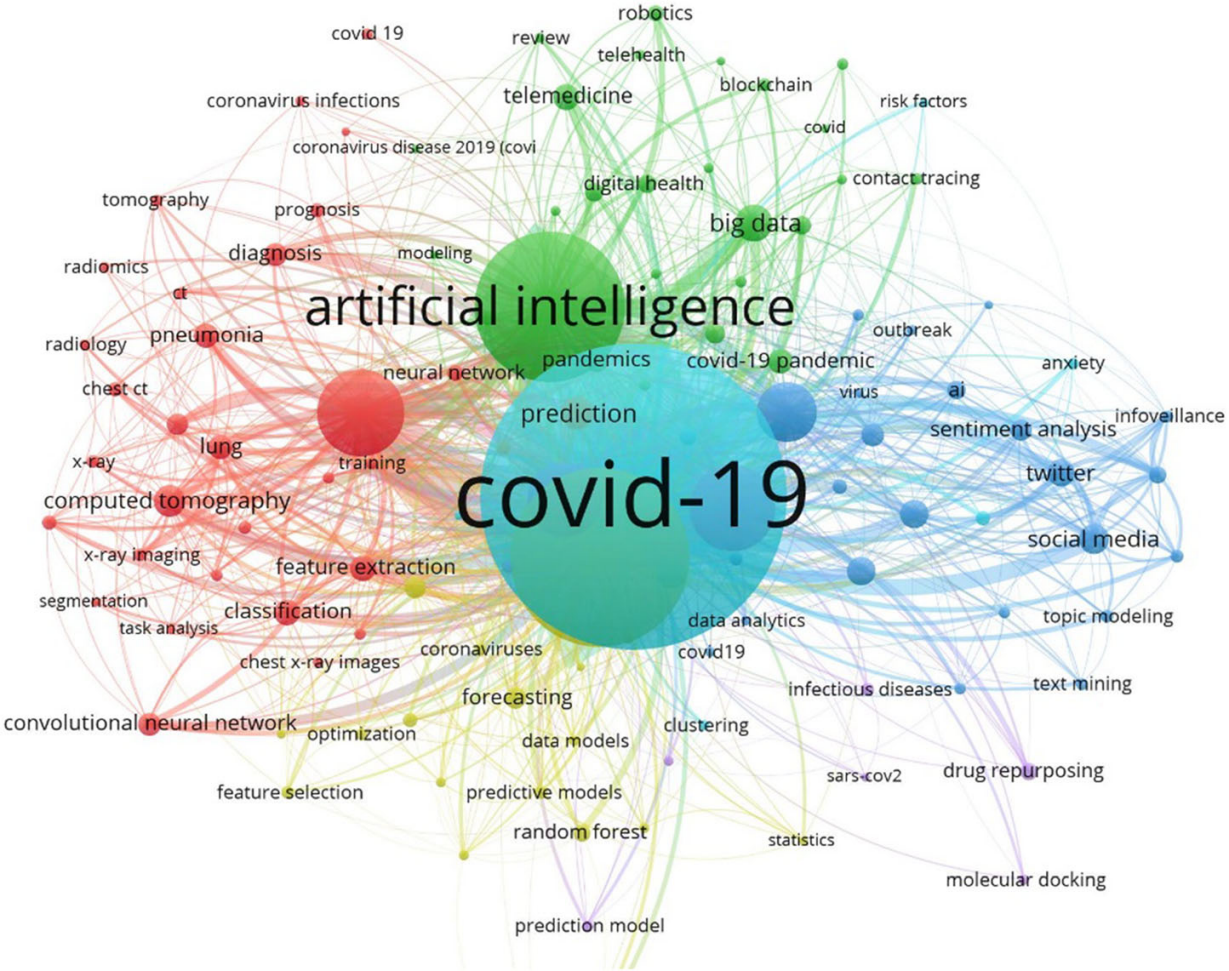
Authors keyword co-occurrence graph

Figure 10 shows the network of co-occurrence of different keywords within a publication, used by various authors. It can be seen that “COVID-19” is the highest occurring keyword in all the publications overall. Width of the curves connecting the nodes in the network demonstrate the frequency of these keywords occurring together within publications. For example, “COVID-19”, “big data”, and “artificial intelligence” have occurred more frequently together when compared to “COVID-19” and “statistics” or “molecular docking” occurring together. Other important keywords in this network are: “machine learning” (not visible in the figure, due to the software’s limitations. It is under the COVID-19 node), “neural network”, “feature extraction”, “classification”, “computed tomography” etc.

### Funding agencies

In addition to the aforementioned results, the agencies that have funded most publications worldwide were also identified during our analysis. This study presented the geographical locations where agencies were generously funding to deal with the pandemic using the AI centric approaches. The top 50 funding agencies worldwide were identified and ranked based on number of publication records funded by them. This is shown in Table 13. The top 5 positions on the list are attained by United States Department of Health Human Services, National Institutes of Health NIH USA, National Natural Science Foundation of China, European Commission, and National Science Foundation that fund 132, 127, 119, 104, and 69 publication records, respectively. In the top 50, USA and China are the countries finding most to the publications related to AI and ML for COVID-19.

**Table 13:** Top 50 worldwide funding agencies

S. No.	Funding Agencies	Publication Records	% share of TP
1	United States Department of Health Human Services	132	5.42
2	National Institutes of Health	127	5.22
3	National Natural Science Foundation of China	119	4.89
4	European Commission	104	4.27
5	National Science Foundation	69	2.84
6	UK Research Innovation	49	2.01
7	Engineering Physical Sciences Research Council	28	1.15
8	Wellcome Trust	21	0.86
9	National Key Research and Development Program of China	20	0.82
10	Conselho Nacional De Desenvolvimento Cientifico E Tecnologico Cnpq	18	0.74
11	National Key R D Program of China	18	0.74
12	Medical Research Council UK	17	0.70
13	Natural Sciences and Engineering Research Council Of Canada NSERC	17	0.70
14	Coordenacao De Aperfeicoamento De Pessoal De Nivel Superior Capes	16	0.66
15	European Research Council	15	0.62
16	Canadian Institutes of Health Research	13	0.53
17	Fundamental Research Funds for The Central Universities	13	0.53
18	King Saud University	13	0.53
19	Ministry of Science and Technology Taiwan	12	0.49
20	National Institute for Health Research	12	0.49
21	Projekt Deal	12	0.49
22	European Commission Joint Research Centre	11	0.45
23	National Natural Science Foundation of Guangdong Province	11	0.45
24	NIH National Cancer Institute	11	0.45
25	NIH National Heart Lung Blood Institute	11	0.45
26	United States Department of Energy Doe	11	0.45
27	Federal Ministry of Education Research	9	0.37
28	NIH National Institute of Allergy Infectious Diseases	9	0.37
29	NIH National Institute of Biomedical Imaging Bioengineering	9	0.37
30	NIH National Institute of General Medical Sciences	9	0.37
31	NIH National Library of Medicine	9	0.37
32	Biotechnology and Biological Sciences Research Council	8	0.33
33	Department of Science Technology India	8	0.33
34	French National Research Agency	8	0.33
35	German Research Foundation	8	0.33
36	Israel Science Foundation	8	0.33
37	Ministry of Science and Technology China	8	0.33
38	Taif University Taif Saudi Arabia	8	0.33
39	China Postdoctoral Science Foundation	7	0.29
40	Chinese Academy of Sciences	7	0.29
41	Department of Biotechnology India	7	0.29
42	Ministry of Education Culture Sports Science & Technology Japan	7	0.29
43	Ministry of Education Singapore	7	0.29
44	Natural Science Foundation of Zhejiang Province	7	0.29
45	NIH National Center For Advancing Translational Sciences	7	0.29
46	Royal Society of London	7	0.29
47	Spanish Government	7	0.29
48	Australian Research Council	6	0.25
49	British Heart Foundation	6	0.25
50	Canada Research Chairs	6	0.25

## Discussion and conclusion

While writing this paper, the entire world is still under the grip of the deadly COVID-19, strying to recover from its devastating impacts during the last two years. To prevent further spread and to gather more insight into the behavior and effect of this deadly virus, researchers from all over the globe, worked desperately to come up with possible vaccines. Biomedical and computational sciences, along with AI and ML have together made this happen in a decent amount of time. This has resulted in a huge volume of scientific publication data, analyzing which, useful insights can be obtained. In this paper for the first time, we have performed an extensive analysis (both qualitative and quantitative) of the AI centric publication related to COVID-19. This analysis has revealed multiple key findings. These are given below:

A. *Detailed key findings*
  Within the last two years, the research on COVID-19 with focus on AI has shown a significant growth. Overall, the papers have gathered 15,607 citations in just 2,434 papers. The papers published in April 2020 have obtained the highest citations till the time this paper is being written, with the count of 542. Notably, there are 85.54% publications which have either no or less than 10 citations count. This is naturally due to the continuous flow of work in the literature. It is anticipated that the citation counts of the quality work will increase with time.
  Apart from this, the maximum number of the papers that have been published are articles, among which many are open access. This is acceptable in the sense that the review process pertaining to these is quicker, and such research work is available to the research community for free. Analyzing the top 50 papers based on citation reveals that most of these have obtained citations between 60 and 100. This reveals the premature status of the AI centric research on COVID-19. Interestingly, there are two papers in the top 50 list which have been published in January and August of 2021 and have gathered significant citations of 124 and 70, respectively. It was also revealed that classification using DL models garnered the highest interest amongst both researchers and readers.
  Amongst the research areas, ‘Computer Science’ and ‘Engineering’ remain at the top, having contributed the most to AI centric research to COVID-19. Similarly, the Journal of Medical Internet Research, IEEE Access, and the International Journal of Environmental Research and Public Health are among the top 3 productive journals for research on AI for COVID-19. On the other hand, the International Journal of Environmental Research and Public Health ranks highest in the most influential journal list, while IEEE Access stands at second spot.
  Considering authors, Al-Turjman published the most number of papers while, Xue obtained the highest citations for research related to AI for COVID-19. Additionally, Harvard Medical School, Huazhong University of Science and Technology, and Chinese Academy of Sciences are the top institutions publishing on COVID-19 focused on AI. However, the University of Oxford stands at the top of the most influential institutions list.
  Country-wise analysis of AI centric research on COVID-19 reveals the top contributors to be USA, China, India, and England. However, Asia is the top continent in terms of both citations and publications. It was also revealed that “COVID-19” is the highest used keyword in all the publications considered. It was coupled the most with keywords “artificial intelligence” and “machine learning”. Also, the United States Department of Health Human Services is the topmost funding agency, which sponsored the research for the highest number of publications.
B. *General observations*
  The current publication data available on AI centric research on COVID-19 is quite significant and growing gradually. Now, the world is in the state of rehabilitation from the havoc of the pandemic. Many AI and ML approaches have been implemented to the COVID data for the data analytics and predictions. Our investigation has revealed some very useful insights related to the AI centric publications on COVID-19. Although most of the research on this topic is recent, publications, in general, have been cited significantly as compared with other matured fields. These numbers will increase over time because AI-centric research on COVID-19 and related studies are still growing in numbers.
  It is interesting to note how the USA and India, being two of the worst hit countries due to COVID-19 in the pandemic, has produced the highest numbers of publications, when considered country-wise. Even though in terms of continents, Asia tops the list on production of papers while also obtaining the highest number of citations. This is an indicator of the fact that multiple countries within Asia are devoted to conducting AI centric research on COVID-19.
  As a future work, we shall include various other indexing platforms such as Scopus, Google scholar and DBLP. Furthermore, we shall extend the work on several factors including institution based bibliometric analysis, country-based analyses, etc. In doing so, we will also target to develop a dynamic platform where we can regularly update the statistics related to the AI centric scientific research on COVID-19.

## Data Availability

Data available on request from the authors.

## Code availability

Code available on request from the authors.
